# Supplementary material for: The interactome of intact mitochondria by cross-linking mass spectrometry provides evidence for coexisting respiratory supercomplexes
Source: Mol Cell Proteomics. 2017 Dec 8;17(2):216–32. doi: 10.1074/mcp.RA117.000470 (PMC5795388; doi:10.1074/mcp.RA117.000470)
Supplement: Supplemental Data [file supp_RA117.000470_133922_0_supp_23978_fzffwf.zip › spectra_annotation/mito_spectra_annotation/47-1-1-1-7-1.pdf]

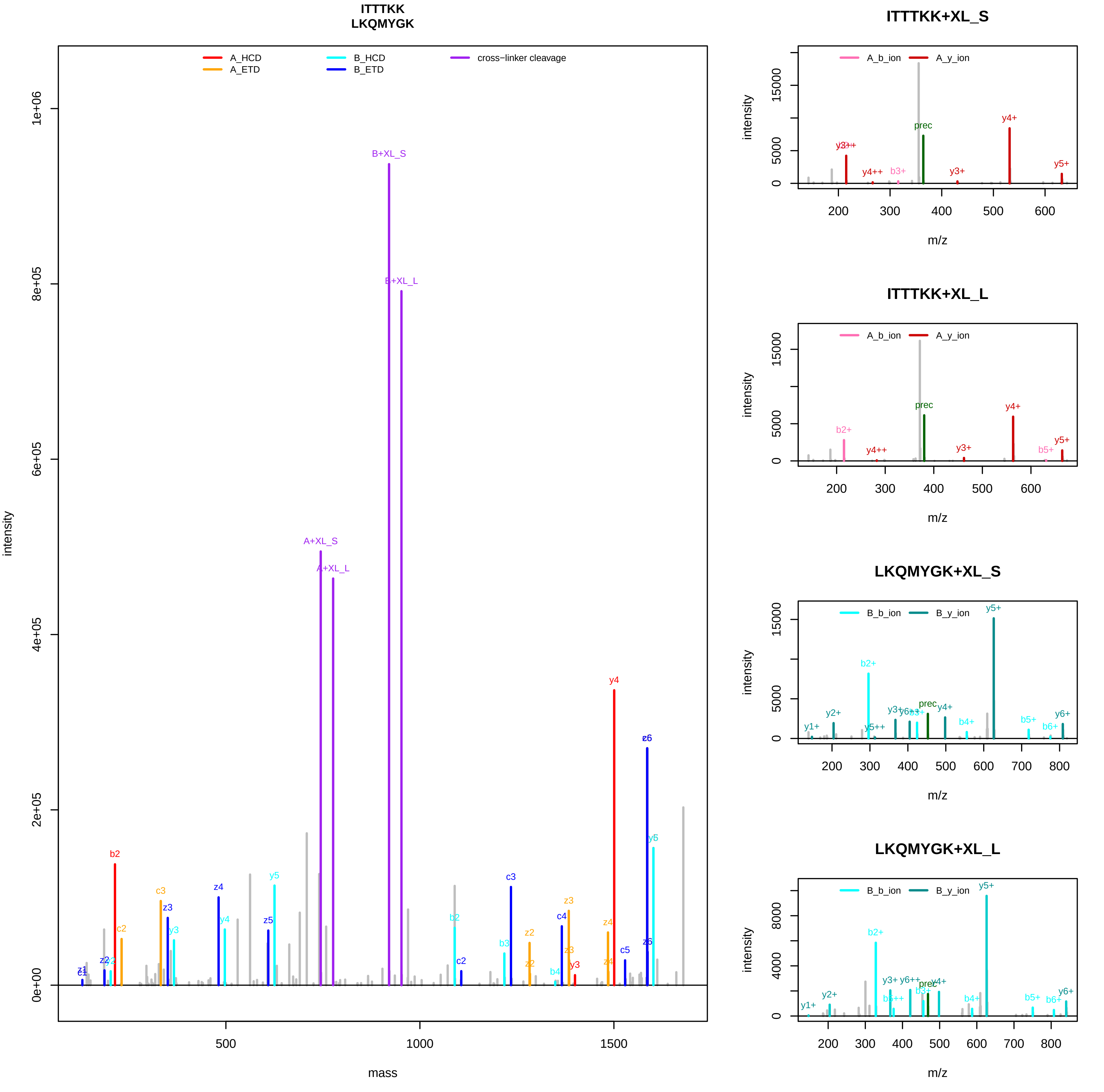
ITTTKK
LKQMYGK
A_HCD
A_ETD
B_HCD
B_ETD
cross−linker cleavage
intensity
mass
0e+00
2e+05
4e+05
6e+05
8e+05
1e+06
500
1000
1500
B+XL_S
B+XL_L
A+XL_S
A+XL_L
b2
c3
c2
z3
y3
z4
y4
z5
y5
b2
c2
c3
b3
z2
c4
z3
y3
z4
y4
c5
z6
y6
ITTTKK+XL_S
A_b_ion
A_y_ion
y3++
y4++
b3+
prec
y3+
y4+
y5+
m/z
ITTTKK+XL_L
b2+
y4++
prec
y3+
y4+
b5+
y5+
LKQMYGK+XL_S
B_b_ion
B_y_ion
y1+
y2+
b2+
y5++
y3+
y6++
b3+
prec
y4+
b4+
y5+
b5+
b6+
y6+
LKQMYGK+XL_L
y1+
y2+
b2+
y3+
y6++
prec
y4+
b4+
y5+
b5+
b6+
y6+
